# The role of cinematic rendering in pre-operative planning of a thoracodorsal artery perforator flap (TDAP) phalloplasty: a case study

**DOI:** 10.1259/bjrcr.20180084

**Published:** 2018-11-22

**Authors:** Carolina Lugo-Fagundo, Hannah Ahn, Devin O’Brien-Coon, Elliot K. Fishman

**Affiliations:** 1 The Russell H. Morgan Department of Radiology and Radiologic Science, Johns Hopkins School of Medicine, Baltimore, MD, USA

## Abstract

The thoracodorsal artery perforator (TDAP) flap is a muscle-sparing skin and fat flap that requires precise intramuscular dissection of the thoracodorsal artery perforators in the axillary region. Pre-operative image-based treatment planning is a crucial part of flap design. In this article, we discuss the first-ever reported use of the cinematic volume rendering technique (CVRT) to evaluate the thoracodorsal artery for a TDAP flap phalloplasty in a 49-year-old transgender patient. Cinematic volume rendering technique uses light maps to generate photo-realistic three-dimensional images of the thoracodorsal artery and its perforators. These images aid the surgeon in evaluating optimal perforators and latissimus dorsi muscle involvement for more efficient flap design.

## Introduction

The thoracodorsal artery perforator (TDAP) flap is a muscle-sparing skin and fat flap that requires careful and precise dissection of the thoracodorsal (TD) artery perforators, which are found deep in the underlying muscle or intermuscular septa.^1^ This flap has gained popularity for its large dimensions and thinness, which facilitates anastomosis and flap insetting during free transfers.^1,2^ It also reduces donor site morbidity from muscle and nerve sparing, decreases post-operative pain and seroma formation, and, unlike the latissimus dorsi flap, does not impair shoulder or arm function.^3–5^ The TD artery and its perforators are the main vessels of the latissimus dorsi muscle, making the dissection of an intramuscular perforator difficult.

Selection of the largest perforator in free flap reconstruction is an important aspect of pre-operative planning, since perforator patency and size determine flap perfusion and survival.^2,6^ It has been reported in the literature that TDAP flaps can be difficult to harvest for a number of reasons, including the presence of few adequate perforators in comparison to other flaps such as the deep inferior epigastric perforator flap, limited knowledge on the distribution and location of the TD artery perforators, and the difficulty in their dissection given their small diameter and intimate relationship with the TD nerve.^1^ To combat these challenges, surgeons adopted an image-based treatment approach in which patients are evaluated using radiological technology such as CT angiography (CTA), Doppler ultrasound or MR angiography (MRA).^7,8^ In this article, we discuss the first-ever reported pre-operative evaluation of a muscle-sparing TDAP flap for phalloplasty using cinematic volume rendering technique (CVRT), a highly realistic three-dimensional (3D) imaging modality. This physically based real-time imaging technique allows for a more realistic assessment of the intramuscular distribution of TD perforators, thus allowing the surgeon to evaluate the viability of the perforator, the risk of flap necrosis, and the potential of a skin-only flap design that reduces donor site morbidity.

## Case report

**Figure 1.**
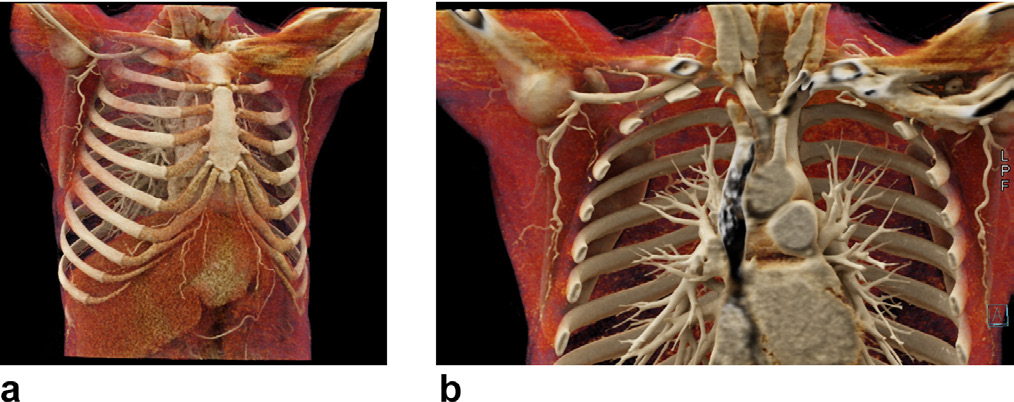
49-year-old transgender patient presented to the hospital for a pre-surgical evaluation of a TDAP phalloplasty. CTA imaging of the chest and evaluation with CVRT revealed well-defined, bilateral thoracodorsal arteries (big arrows) and its perforators (small arrows). (A) Right anterior oblique projection view. (B) Anterior projection view. CTA, CT angiography; CVRT, cinematic volume rendering technique; TDAP, thoraco dorsal artery perforator.

A 49-year-old transgender patient presented to the Johns Hopkins Outpatient Center for pre-surgical evaluation for phalloplasty (Figure 1). A phalloplasty is a female-to-male urogenital gender affirmation surgery performed in transgender individuals.^9^

The construction of a new phallus is most commonly achieved through the harvest of a flap. The musculocutaneous latissimus dorsi (MLD) flap, one of several donor sites that can be used in the reconstruction of the phallus, spares most of the latissimus dorsi muscle; the pedicle is dissected proximally until the origin of the subscapular artery can be identified.^2^

**Figure 2.**
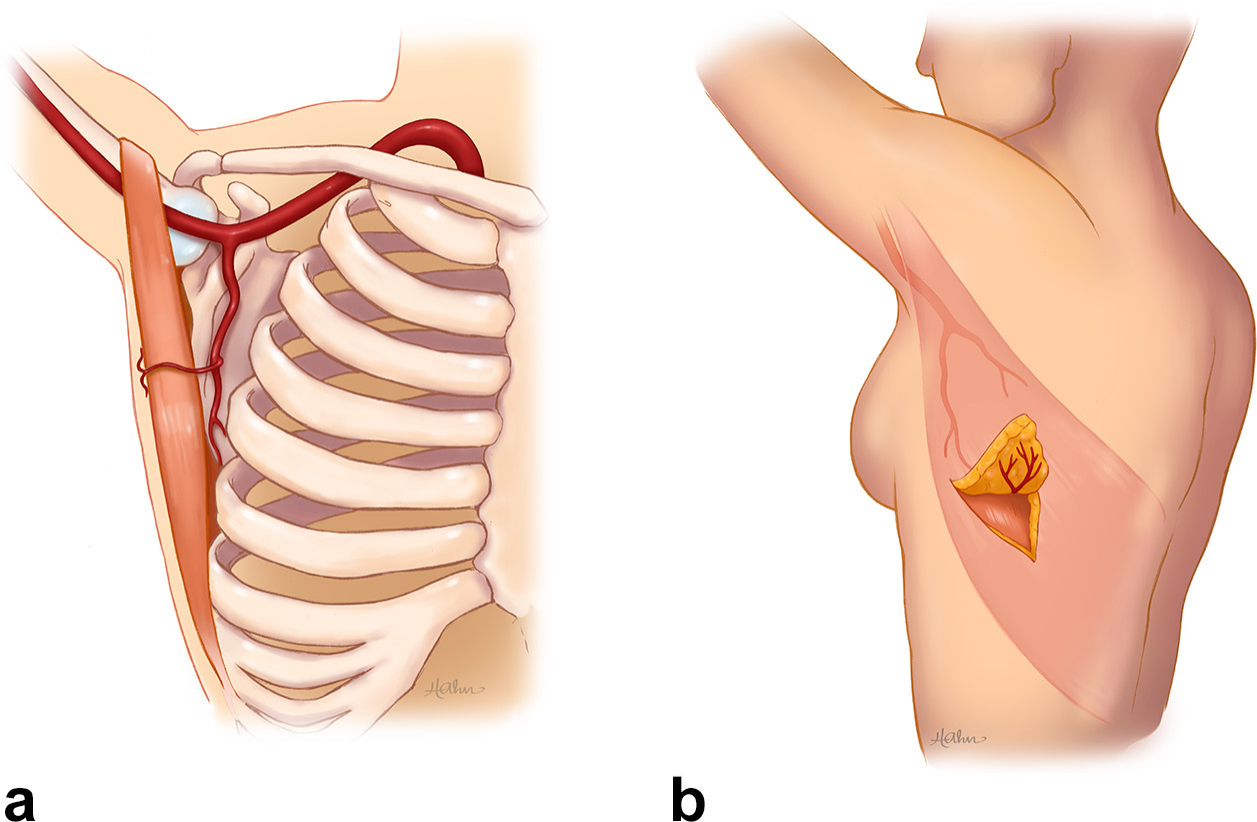
Illustrations of the thoracodorsal artery as viewed in CVRT (a) and surgery (b).

Rather than create a standard MLD, we sought to perform a TDAP flap that would not sacrifice muscle while yielding lower morbidity of latissimus function and a less bulky phallus. The TDAP has been used for various applications; however, there is no American literature that reports on its implementation in phallic reconstructions.^10^ The challenge of harvesting and supplying this large muscle-sparing flap with a single perforator lies in identifying the presence of a large appropriate perforator and placement of the flap design over it (Figure 2). As a result, CT angiography has gained increasing popularity for pre-operative deep inferior epigastric artery perforator mapping.^4^

Therefore, the attending physician ordered a CTA. During this study, the patient was asked to lie in the supine position with his arms extended over the head in order to image the TD artery and its perforators. The patient was injected with 100 cc of omniopaque −350 at 4 cc s^–1^ with the injection in the contralateral extremity. Delayed images were obtained to help minimize the artefact from contrast and venous structures on a left-sided injection. 3D images were obtained with CVRT.

The 3D mapping demonstrated well-defined bilateral TD arteries and perforator branches. CVRT produced a detailed and realistic anatomical representation of the patient’s thoracodorsal artery. The 3D reconstructed images were manipulated such that different tissue types and anatomical structures were highlighted, allowing for a more in-depth understanding of the patient’s anatomy. This optimal view of the anatomy is crucial in pre-operative planning of TDAP flaps, since the anatomy of the TD artery and its perforators can vary greatly in relation to the latissimus dorsi muscle. It also provides the surgeon with more information to design the flap and to plan for a safe and efficient surgery. From the study, the physician was able to judge the location and anatomical distribution of the TD artery perforators and their course in relation to adjacent muscle.

## Discussion

The MLD flap is a flap procedure commonly used in phalloplasty.^11^ However, the use of TDAP flaps has never been reported in American literature. Though an appealing option for a phalloplasty given its thinness, large size, sparing of the latissimus dorsi muscle, and less bulky appearance in the neophallus, the large TDAP flap poses the risk of necrosis in the absence of a single large TD perforator. Pre-operative evaluation of the perforators is required in order to assess the potential of using a perforator or muscle flap. Pre-operative image-based planning is useful for determining location and size, as well as intramuscular, subfascial, and subcutaneous segments of each of the TD artery’s perforators.^6^ Patients who have perforators that are too small or that microscopically lack tissue blood perfusion can be identified through imaging and thus treated appropriately with a muscle flap rather than a perforator flap.^12^ On the other hand, the selection of the largest perforator through the use of imaging technology, such as Doppler ultrasound, CTA or MRA, in pre-operative planning of patients who qualify for a skin-only flap decreases donor site morbidity, increases surgeon comfort level, reduces intraoperative stress and procedure time, and prevents partial flap loss, flap necrosis, and fat necrosis.^4,6,12^ Color Doppler ultrasonography may also be used by experienced surgeons in pre-operative planning of TDAP flap-based reconstructions; however, this technique is observer dependent.^13^

The use of CVRT in pre-operative planning, specifically for the evaluation of the TD artery in a potential TDAP flap phalloplasty, has never been reported in the literature. CVRT uses different light maps to generate a realistic depiction of medical data.^14^ It displays 3D reconstructed volumetric CT data by using a much more complex ray-casting projection model that is lacking in traditional volume-rendering techniques.^15^ CVRT reproduces surface-rendered 3D images collected from all voxels encountered in a projection ray, providing spatial information that is absent in conventional CT images.^15^ CVRT features improved perception of depth and soft tissue structures, allowing it to display a more photo-realistic representation of anatomical regions, and facilitating interpretation by not only the radiologist but also the surgeon.^14^ In order to optimize anatomical views, the surgeon can modify the display options as with any 3D multidetector CT reconstruction method.^15^ CVRT easily highlights structures with the highest Hounsefield units such as the TD artery in this case; however, display settings can be modified to study and evaluate the close relationship of the vessel with the latissimus dorsi muscle. The superior display of 3D volume-rendering images allows the surgeon to easily and independently visualize the intramuscular course and distribution of the TD perforators and to assess the potential of a skin-only perforator flap as opposed to a muscle-harvesting flap. This pre-surgical decision-making process allows for a safer dissection and a more predictable surgical outcome.

## Conclusion

CVRT is a useful imaging technique for pre-operative evaluation of flap designs. It allows the surgeon to assess vessel versatility via a 3D photo-realistic representation of the anatomical distribution of the perforators relative to the muscle and other structures around it. This allows for efficient evaluation of a TDAP flap, which is associated with superior surgical outcomes. However, future studies should be conducted in order to further assess the value and use of CVRT in perforator flap design.

## Learning points

- Imaging with CTA and evaluation with CVRT provide an optimal view of the TD artery in relation to surrounding anatomical structures.
- Pre-surgical imaging of the TD artery allows for pre-operative decision making on skin-only or muscle flaps.
- Pre-operative planning provides the patient with best surgical outcomes by anticipating complications and suggesting the most favorable surgical technique.
